# Medicine

**Published:** 1904-09

**Authors:** G. Parker


### Progress of the Medical Sciences.

### MEDICINE.

**Ateleiosis and Progeria** are names given to opposite types of disorder in the development of the body. Prolonged infantilism or premature old age are met with under various conditions. Thus they may be merely slight variations from the normal rate of progress. They may occur as symptoms in certain diseases such as syphilis, but in the types under consideration the abnormality is definite and extreme, though not connected with any known disease. Cases of ateleiosis have been described by Mr. Hastings Gilford,[1] and some were shown by him at Oxford. They may be divided into two classes. In the asexual one the individual remains permanently a child in size and appearance, and though the development varies in different parts of the body, the sexual organs are the most backward of any. Thus a boy of twelve showed epiphysial ossification which would be normal at six years, features and a skull like those of a child of three, the height of a boy of five or six years, the dentition of eleven years, and infantile sexual organs.

In a second group there is a similar arrest of development until puberty, when the epiphyses unite and the sexual organs mature, so that the individual remains in general appearance and height a child, but sexually is an adult. A middle-aged patient and his son aged ten shown at Oxford were both under thirty-eight inches high. The condition seems compatible with perfect health in other respects, and is not connected with any known disease, nor can any cause be assigned to it. In marked contrast to these cases are the instances of sporadic cretinism,

[1] *Med.-Chir. Trans.*, 1902, lxxxv. 305; *Practitioner*, 1903, lxx. 797.

morbus cæruleus, Mongolian idiocy, and perhaps what Byrom Bramwell terms "pancreatic infantilism." In the latter the growth of the body ceases, and a chronic diarrhœa with swollen and tympanitic abdomen are noticeable, but the patient may be mentally bright and intelligent. The epiphyses remain ununited and the sexual organs are infantile.

The absence of the pancreatic secretion in one of these patients was proved by David Young from (1) the excess of fat in the stools, (2) the small amount of phosphoric acid in the urine on a milk diet, and (3) the results of Sahli's test. Under the administration of a glycerine extract of the pancreas rapid growth, cessation of diarrhœa, and sexual maturity took place and the other symptoms ceased.[1]

Only two certain cases of progeria are recorded by Mr. Gilford, one of which died at seventeen and the other at eighteen with the marks of old age, lean, feeble, decrepit, and bald. The skin was dry and wrinkled; some of the arteries were calcareous, and in one at least there was a persistent thymus. Both were under 40 inches in height, and showed a "curious mixture of immature development on the one hand and of premature old age on the other." The disease probably commenced about the period of the first dentition, and is not a termination of ateleiosis. The writer suggests that acromegaly and progeria may be opposite disorders.[2]

The *Practitioner*, in speaking of these cases, refers to the death in 1754 of Hopkins Hopkins, weighing seventeen pounds at most, with all the marks of old age, though only seventeen years old. Portraits of some of Mr. Gilford's patients are published in the volume of the *Transactions of the Medico-Chir. Society* already cited.

**The Early Diagnosis of Phthisis.** Much has been written of late on this subject because it is strongly felt that to obtain a high percentage of cures, and not merely success in favourable cases, the treatment must be commenced far sooner than is usually done. If we are starting sanatoria great care is needed to choose from the crowd of applicants those patients who will probably get lasting benefit. Thus C. H. Cattle[3] would select only: (1) Cases with the disease limited to one lobe of one lung without signs of cavity, and whose temperature is not much above normal after a week or two of rest in bed; (2) Many cases of pleurisy on account of their frequent lapse into phthisis. He would exclude acute cases unless the symptoms subside without great destruction of lung, all in which a cavity can be recognised except some chronic cases in which considerable repair has occurred, and all cases with glycosuria, tuberculosis

[1] *Scottish M. & S. J.*, 1904, xiv. 321.
[2] *Med.-Chir. Trans.*, 1897, lxxx. 17; *Practitioner*, 1904, lxxiii. 188.
[3] *Practitioner*, 1904, lxxii. 218.

of the kidney, or intestinal ulceration. Cases with feeble circulation and frequent bronchial catarrh may often be better treated abroad.

What then are the earliest symptoms of phthisis? F. S. Clarke[1] emphasises the following points: (1) An increased pulse-rate; (2) Gradual loss of weight without an obvious cause; (3) Digestive disturbances; (4) An evening rise of temperature in some cases; (5) Tubercle bacilli, if present in considerable numbers; (6) Vesiculo-bronchial breathing; (7) Small crepitant rales at the end of inspiration; and in addition we may have recourse to the tuberculin test or to the X-rays for confirmation. Some few of these symptoms may be all we have to guide us if the disease is to be checked at once. As Cattle says, the patient may complain of nothing pointing to chest trouble, but only of dyspepsia or lassitude. Yet inquiry may show a history of hæmoptysis, pleurisy, or probable contagion, and the rectal temperature may be found to be abnormal after exertion. There is in these early days often an absence of bacilli, and much time may be lost in relying upon negative reports, though when no physical signs exist, the sudden expectoration of a lump of purulent sputum laden with bacilli may lead to the fortunate discovery of hidden mischief. Suspicion may be raised by the general appearance, or the shape of the chest. This is usually described as flat or alar, and when seen with the long neck and prominent larynx, so dear to some artists, and the hair growing down the back, it has long been held of evil augury. Woods Hutchison, however, shows that in phthisical patients the antero-posterior diameter is not generally reduced. The ratio in health of the two diameters in adults is as 100 to 70, whereas in forty tubercular cases in America he found the ratio was 100 to 79.5. At Brompton it was 100 to 79.6, and Colbeck at Victoria Park found it was 100 to 80.3. Thus something more than the actual ratios must be considered if any reliance is to be placed on the shape. As to physical signs, there are two causes which prevent our getting much aid from them in early stages of the disease, as Isambard Owen[2] points out. One is the essentially intermittent character and progress of the complaint, so that they may not be apparent at the particular time of the examination. The other is the fact that most of the signs are given only by lesions near the surface of the lung. Among other difficulties we may notice Cattle's point, that in health we so frequently hear harsh breathing and increased vocal resonance at the right apex that when they occur as a result of disease they are apt to be overlooked. Increased resistance to percussion and diminished movement at the apex may precede dulness or other signs or cough. Sometimes early and very important signs are found in diminished breath sounds over the right scapula, or

[1] *Med. Rec.*, 1904, lxv. 55. [2] *Brit. M. J.*, 1904, i. 765.

breath sounds at its angle which are only equal to or less than those on the opposite side, whereas they ought to be louder. After rickets and similar conditions an asymmetry of the chest may be found, which not only makes the heart itself appear abnormal and displaced but may give rise to misleading differences in the percussion note.[1] Persistent rales at the end of inspiration in the upper part of the lungs are among the most reliable signs we have, but they must be distinguished from the dry, dull crackle of apical emphysema (Isambard Owen) and the bubbling heard during the swallowing of frothy saliva (Baumler). Moreover, these rales may be found only towards the axilla, or on the upper part of the first interscapular space on the right side, instead of at the apex. Fine rales along the border of the left lung with a history of hæmoptysis have often been put down to phthisis, while their real cause was mitral stenosis. It has been said that the true rales of phthisis may be heard by Cybulski's method of listening with the observer's ear about three inches from the patient's open mouth, and Remouchamps thinks[2] that fine crepitations of a particular type heard in this way are pathognomonic, and indeed one of the best tests we have.

Hæmoptysis again is a most important indication if we can be sure of its origin. Though there may be no physical signs to supplement it a careful watch should be made whenever it occurs, for symptoms such as pyrexia, or loss of weight, or the presence of bacilli. Sometimes the X-rays will show marked changes in the upper part of the lungs or altered movements, which may aid our diagnosis. Thus Stanley Green, at the Clinical Society in May, spoke of the unilateral check to the movements of the diaphragm, the presence of scattered areas of shadow, and changes in the slope and shape of the ribs as revealed in certain cases by the rays.

**Serum Diagnosis.** The tuberculin test is comparatively little used in this country. Koch, however, claimed that in 3,000 cases he had positive results in 99 per cent. Occasional failures do, however, occur. Thus sometimes reactions are seen in syphilis. Mazziotti[3] notes them as occurring in convalesence after typhoid, and possibly in some cases of chlorosis. Arloing devised an agglutination method with pleural fluid, and much discussion has taken place as to its value. If any high degree of accuracy is to be obtained a constant amount of the fluid and of cultures of known strength must be used, as Buard points out,[4] and the time required must be fixed. Cyto-diagnosis is another procedure based on the enumeration of the cells in pleural effusions, brought forward by Widal and Ravaut. In tubercular cases they found the cells were chiefly lymphocytes with some red cells, and never more than 10 per cent. of them

[1] Baumler, *Ibid.*, 769. [2] *Semaine méd.*, Dec. 2, 1903, p. 392.
[3] *Clin. med. Ital*, No. 9, 1901.
[4] *Gaz. hebd. d. Sc. méd. de Bordeaux*, 1901, xxii. 351.

were polynuclear ones, whereas in pneumococcal and similar cases the polynuclear cells were in the majority. However, H. C. Earl[1] remarks that in the early stage of tubercular cases, *i.e.* for the first ten days, polynuclear cells are very numerous, while in the effusions of more or less advanced phthisis the cells may vary, possibly because a mixed infection exists at that time. With these exceptions, he thinks that the test is a reliable one. Under certain conditions the serum from joints affords similar indications, and in most cases of tubercular meningitis there is also a marked increase of lymphocytes in the cerebro-spinal fluid, though this is found also in tabes, general paralysis, and some other states. Though these tests are not yet completely understood, they are capable of giving useful information in some cases, and the examination of pleural fluid is especially important.

G. Parker.

